# Regional brain volumes in brief psychotic disorder

**DOI:** 10.1007/s00702-020-02140-y

**Published:** 2020-01-18

**Authors:** Hua Li, Szabolcs Kéri

**Affiliations:** 1 National Institute of Psychiatry and Addictions, Budapest, Hungary; 2 Department of Cognitive Science, Budapest University of Technology and Economics, Egry J. str. 1, 1111 Budapest, Hungary; 3 Department of Physiology, University of Szeged, Szeged, Hungary

**Keywords:** Brief psychotic disorder, Schizophrenia-spectrum, Magnetic resonance imaging, Frontal cortex

## Abstract

Brief psychotic disorder (BPD) is a relatively rare representative of psychotic disorders. Structural brain abnormalities in BPD are not known. We compared 30 patients with BPD and 30 matched healthy controls using high-resolution structural T1-weighted magnetic resonance imaging (MRI). We performed cortical/subcortical reconstruction and volumetric segmentation using FreeSurfer v6.0. Results revealed that the caudal/rostral middle frontal cortex, superior frontal cortex, and the frontal pole were significantly smaller in patients with BPD compared to controls. The number of lifetime psychotic episodes negatively correlated with caudal middle frontal and frontal pole volumes. These results indicate structural abnormalities of the frontal cortex in BPD, which are associated with the number of psychotic relapses.

## Introduction

Mounting evidence from research conducted over the past decades suggests that schizophrenia is characterized by multifocal cortical and subcortical structural abnormalities (Fornito et al. 2009; Haijma et al. 2013; Honea et al. 2005; Shepherd et al. 2012; Wright et al. 2000). Although early meta-analyses indicated smaller mean cerebral volume, ventricular enlargement, and regional volume reductions in the medial-temporal cortex (hippocampus/amygdala, parahippocampal region) in patients with schizophrenia relative to healthy control subjects (Wright et al. 2000), more recent analyses uncovered grey matter reductions in an extended network including frontal, temporal, thalamic, and striatal regions (Fornito et al. 2009).

Much less is known about potential brain anomalies in brief psychotic disorder (BPD), a rare representative of psychotic disorders (Marneros and Pillmann 2004). Some symptoms of BPD are similar to those seen in schizophrenia (delusion, hallucinations, disorganized speech, and grossly disorganized or catatonic behavior), but clinicians often observe a sudden onset and a short duration of symptoms (at least 1 day, but less than 1 month) (American Psychiatric Association 2013). Most of the patients with BPD make a full recovery, their psychosocial functions are relatively spared, and negative symptoms are missing or minimal. Meta-analytic evidence suggests a better long-term prognosis of BPD compared with remitted first-episode schizophrenia (Fusar-Poli et al. 2016a). The prospective diagnostic stability of BPD is clinically acceptable, but less stable than that of schizophrenia (Fusar-Poli et al. 2016b). Interestingly, several individuals with Brief Limited Intermittent Psychotic Symptoms (BLIPS), a high-risk state of long-lasting psychosis, meet the criteria of Acute and Transient Psychotic Disorder (ATPD). Many of these patients develop schizophrenia-spectrum disorders, and therefore BPD is being used to identify patients at risk of developing persistent psychoses (Fusar-Poli et al. 2017a, b, c; Singh et al. 2004). The etiology and epidemiology of BPD are not clear, although severe acute stress, trauma, recent childbirth, immigrant status, and living in a developing country are among the risk factors (Castagnini and Fusar-Poli 2017).

The paucity of neurobiological findings in BPD is a major shortcoming in the literature (Castagnini and Fusar-Poli 2017; Malhotra et al. 2019). Therefore, to explore structural brain abnormalities in BPD, we recruited 30 patients with BPD in clinical remission and 30 matched healthy controls. Based on previous results from other psychotic disorders (Fornito et al. 2009; Haijma et al. 2013; Honea et al. 2005; Shepherd et al. 2012; Wright et al. 2000), we hypothesized that patients with BPD exhibit smaller regional volumes in neocortical and limbic areas with special reference to frontal and temporal regions, including the hippocampal formation, relative to control participants. In addition, we explored the correlations between regional brain volumes and clinical characteristics (illness duration, number of lifetime psychotic episodes, and cumulative antipsychotic medication).

## Methods and materials

### Participants

We enrolled 30 outpatients with BPD and 30 healthy control subjects matched for age, gender, education, general psychosocial functions, and body-mass index (BMI) (Table 1). The study was coordinated in the Nyírő Gyula National Institute of Psychiatry and Addictions (NIPA) (Budapest, Hungary, 2013–2019), and the BPD patients were recruited at the psychosis outpatient unit of this Institute. For the clinical assessment, we used the Structured Clinical Interview for DSM-5 Disorders—Clinician Version (SCID-5-CV) (First et al. 2016), the Positive and Negative Syndrome Scale (PANSS) (Kay et al. 1987), and the WHO Disability Assessment Schedule (WHODAS 2.0) of the DSM-5 (American Psychiatric Association 2013). BPD was diagnosed according to the DSM-5 criteria: “A. Presence of one (or more) of the following symptoms. At least one of these must be (1), (2), or (3): 1. delusions, 2. hallucinations, 3. disorganized speech (e.g., frequent derailment or incoherence), 4. grossly disorganized or catatonic behavior. (Note: Do not include a symptom if it is a culturally sanctioned response.); B. Duration of an episode of the disturbance is at least 1 day but less than 1 month, with eventual full return to premorbid level of functioning. C. The disturbance is not better explained by major depressive or bipolar disorder with psychotic features or another psychotic disorder such as schizophrenia or catatonia and is not attributable to the physiological effects of a substance (e.g., a drug of abuse, a medication) or another medical condition.” (American Psychiatric Association 2013). Family history and premorbid functioning were rated on the OPCRIT + system (Rucker et al. 2011). We used the WAIS-III for the assessment of general intellectual and cognitive functioning (Wechsler 1997). The scales and interviews were administered by trained clinical psychologists or psychiatrists. The interrater reliability values for the clinical scales were very good or excellent (Fleiss’ $\kappa$  > 0.8).

**Table 1:** Demographic and clinical characteristics

	Brief psychotic disorder (BPD) (*n* = 30)	Healthy controls (*n* = 30)
Male/female	11/19	11/19
Age (years)	39.6 (7.2)	39.1 (7.0)
Education (years)	12.5 (4.8)	12.3 (3.9)
Wechsler Adult Intelligence Scale-III (IQ)	106.4 (12.5)	105.9 (11.7)
Number of tobacco smokers	11	11
Family history of psychiatric disorders		
Any psychiatric disorder	6 (20%)	7 (23.3%)
Schizophrenia	0	0
Affective disorder	3 (10%)	4 (13.3%)
Body-mass index (BMI)	25.3 (3.2)	25.2 (3.7)
WHO Disability Assessment Schedule 2.0 (WHODAS 2.0)	13.1 (4.0)	13.3 (3.9)
Premorbid functioning		
Poor premorbid work adjustment	4 (13.3%)	–
Poor premorbid social adjustment	4 (13.3%)	–
Premorbid personality disturbance	6 (20%)	
Duration of illness (years)	12.5 (6.6)	–
Number of lifetime psychotic episodes	4.5 (2.9)	–
Positive and Negative Syndrome Scale (PANSS)		
Positive	8.7 (2.7)	–
Negative	8.1 (3.0)	–
General	19.7 (5.3)	–
Lifetime antipsychotic dose-years	4.6 (2.3)	–

Data are mean (standard deviation) except for the male/female ratio and the number of tobacco smokers. The two groups did not differ in gender ratio, age, education, BMI, and WHODAS 2.0 (*p*s > 0.5)

We applied the following exclusion criteria: neurological disorders, history of personality disorders and mental disorders other than BPD, evidence of head injury and trauma based on self-reports and medical records, and lifetime psychoactive substance misuse confirmed by the clinical history or by a urine test. There was no evidence of brain injury on the MRI scans (e.g., encephalomalacia). All patients were in clinical remission according to the Andreasen-criteria (Andreasen et al. 2005). Eight patients received second-generation antipsychotic medications at the time of testing [olanzapine, quetiapine, and aripiprazole; chlorpromazine-equivalent dose: 268 mg/day (SD = 194.3)] (Leucht et al. 2015). We gathered data on the lifetime use of antipsychotics by reviewing the comprehensive inpatient and outpatient medical records. The lifetime use of antipsychotic medications was defined as the dose-years of a daily dose of 100 mg chlorpromazine equivalent (Husa et al. 2014). Given that most of the patients (*n* = 28) received second-generation antipsychotics during their previous treatments, we did not calculate first- and second-generation drugs separately. The study was approved by the Hungarian Scientific and Research Committee of the Medical Research Council, Budapest, Hungary. All participants gave written informed consents.

### Structural brain imaging

T1-weighted, high-resolution scans were obtained at the imaging facility of the NIPA (Budapest) (Philips Achieva 3 T scanner, MPRAGE [magnetization-prepared rapid acquisition gradient echo], 3D sagittal acquisition, square field of view (FOV) = 256 mm, acquisition matrix: 256 × 256, voxel size: 1 × 1 × 1 mm^3^, TI = 900 ms, TE (shortest) = 3.16 ms, flip angle: 9 degrees, no fat suppression, full k space, no averages, acquisition time: 6 min and 50 s, acceleration factor: 2). Image processing was performed with Free-Surfer v6.0 (Athinoula A. Martinos Center for Biomedical Imaging). An extensively used, standard, and open protocol was implemented, as described in detail in several previous studies (https://surfer.nmr.mgh.harvard.edu/.) (Fischl 2012). All scans were visually inspected for motion artifacts before FreeSurfer analysis. No scans had to be excluded because of motion artifacts. Brain regions were defined according to the Desikan-Killiany atlas (Desikan et al. 2006). In addition to intracranial volume and total gray/white matter volume, this atlas enables the measurement of 32 regional cortical volumes and 8 subcortical volumes. To minimize the number of variables (type I errors due to multiple comparisons), the volumes of each brain region in the left and right hemisphere were summed, and the total volume of each region (altogether 43 variables) was included in the statistical analysis.

### Statistical analysis

We used STATISTICA 13.0 (Tibco, Palo Alto) and JASP (version 0.9.2., JASP Team) softwares for data analysis. Means and standard deviations were calculated for the variables of interest (regional brain volumes). We applied Kolmogorov–Smirnov tests (normality of distribution) and Levene’s tests (homogeneity of variance). One-way analyses of variance (ANOVAs) were performed on each regional brain volume to compare patients with BSD and healthy controls. The ANOVAs were repeated using a Bayesian approach. Pearson’s product–moment correlation coefficients (*r*s) were calculated between brain volumes and clinical characteristics. To test the potential confounding and the modulating effect of age, education, social and occupational status, clinical symptoms, and BMI, analyses of covariance (ANCOVAs) were used. To minimize the likelihood of type I errors, multiple comparisons were corrected with the Benjamini–Hochberg False Discovery Rate (FDR) method. The level of statistical significance was set at  < 0.05.

## Results

### Differences in brain volumes

Table 2 depicts the regional brain volumes in patients with BPD and healthy control subjects. We found significantly smaller regional brain volumes in patients with BPD relative to control subjects in the caudal middle frontal region [*F*(1,58) = 9.10, *p* < 0.005, BF_10_ = 10.0], rostral middle frontal region [*F*(1,58) = 14.19, *p* < 0.001, BF_10_ = 68.9], superior frontal cortex [*F*(1,58) = 12.38, *p* < 0.002, BF_10_ = 35.4], and the frontal pole [*F*(1,58) = 14.33, *p* < 0.001 BF_10_= 72.7]. There were no significant differences in the remaining brain areas (*p*s > 0.2, BF_10_ < 1) (Table 2). The results remained the same when total brain volume, age, education, BMI, PANSS, and SOFAS scores were included separately in the analyses as covariates. There were no significant differences between male and female patients with BSD (*p* > 0.5).

**Table 2:** Regional brain volumes in patients with brief psychotic disorder and healthy control subjects

Bilateral brain region	Brief psychotic disorder (*n* = 30)	Healthy controls (*n* = 30)	*F*	*p*	*d*
Intracranial volume	1,456,552.3 (29,746.3)	1,457,125.1 (28,421.5)	0.01	0.94	0.02
Cortical gray volume	497,428.6 (6370.1)	497,834.3 (6243.7)	0.06	0.80	0.07
Cortical white matter	503,472.6 (9170.6)	503,684.9 (9003.4)	0.01	0.93	0.02
Caudal anterior cingulate	4320.0 (270.5)	4380.2 (254.3)	0.78	0.38	0.23
Caudal middle frontal*	13,173.1 (780.2)	13,730.5 (650.7)	9.10	0.003 (0.03)	0.80
Cuneus	6310.1 (260.3)	6328.1 (257.5)	0.07	0.79	0.07
Entorhinal	3861.5 (148.3)	3858.7 (146.4)	0.005	0.94	0.02
Fusiform	20,205.4 (628.5)	20,194.1 (631.3)	0.005	0.94	0.02
Inferior parietal	29,778.4 (763.2)	29,883.7 (758.5)	0.29	0.59	0.14
Inferior temporal	22,540.2 (602.2)	22,560.6 (597.0)	0.02	0.89	0.03
Lateral occipital	24,570.4 (554.6)	24,563.6 (559.3)	0.002	0.96	0.01
Lateral orbital frontal	15,700.5 (649.8)	15,830.0 (660.2)	0.59	0.47	0.20
Lingual	14,111.2 (632.5)	14,104.9 (641.0)	0.001	0.97	0.01
Medial orbital frontal	10,835.6 (257.7)	10,947.3 (249.4)	2.91	0.09	0.45
Middle temporal	24,680.7 (597.3)	24,520.6 (601.0)	1.07	0.31	0.27
Parahippocampal	4597.0 (245.7)	4621.5 (239.2)	0.15	0.70	0.10
Paracentral	7760.3 (281.2)	7810.0 (235.4)	0.55	0.46	0.20
Pars opercularis	9554.6 (392.9)	9570.5 (380.0)	0.03	0.87	0.04
Pars orbitalis	4967.3 (202.5)	5074.6 (204.5)	4.17	0.05	0.52
Pars triangularis	8237.7 (246.3)	8300.7 (234.8)	1.03	0.31	0.27
Pericalcarine	4100.6 (270.4)	4150.6 (257.3)	0.54	0.47	0.19
Postcentral gyrus	19,663.5 (516.2)	19,574.1 (464.9)	0.50	0.48	0.19
Posterior cingulate	6762.6 (340.0)	6890.9 (325.5)	2.23	0.14	0.38
Precentral	27,670.3 (645.4)	27,690.2 (632.6)	0.01	0.90	0.03
Precuneus	21,055.1 (464.3)	21,198.7 (496.2)	1.34	0.25	0.30
Rostral anterior cingulate	5103.8 (250.2)	5178.5 (246.3)	1.36	0.25	0.30
Rostral middle frontal*	32,868.0 (768.8)	33,554.3 (691.6)	14.19	0.0004 (0.009)	0.95
Superior frontal*	44,955.0 (682.3)	45,541.2 (605.9)	12.38	0.0009 (0.01)	0.93
Superior temporal	28,452.4 (605.6)	28,500.3 (614.2)	0.09	0.76	0.08
Supramarginal	22,430.2 (663.5)	22,501.9 (657.2)	0.18	0.68	0.11
Frontal pole*	1350.7 (123.6)	1467.6 (115.3)	14.33	0.0004 (0.009)	0.98
Temporal pole	5200.4 (203.3)	5194.5 (208.5)	0.01	0.91	0.03
Transverse temporal	2190.5 (205.5)	2194.7 (204.3)	0.006	0.94	0.02
Insula	17,440.0 (458.0)	17,493.3 (446.2)	0.21	0.65	0.12
Lateral ventricles	13,002.6 (962.0)	12,945.5 (836.1)	0.06	0.81	0.07
Cerebellum	119,445.4 (3890.6)	119,654.4 (3791.0)	0.04	0.83	0.06
Thalamus	15,022.3 (376.2)	15,011.4 (380.0)	0.01	0.91	0.03
Caudate	7246.1 (218.3)	7220.0 (209.4)	0.22	0.64	0.12
Putamen	10,315.3 (296.3)	10,295.6 (301.7)	0.07	0.80	0.07
Pallidum	3235.1 (247.4)	3194.7 (265.9)	0.37	0.55	0.16
Hippocampus	8867.3 (429.3)	9001.4 (355.5)	1.73	0.19	0.34
Amygdala	3793.2 (345.3)	3760.2 (352.2)	0.13	0.71	0.10
Accumbens	1343.3 (299.6)	1332.0 (265.0)	0.02	0.88	0.04

Data (mm^3^) are mean (standard deviation)

*Significant between-group differences (FDR corrected *p* values are shown in parentheses)

### Brain volumes and clinical characteristics

**Fig. 1:**
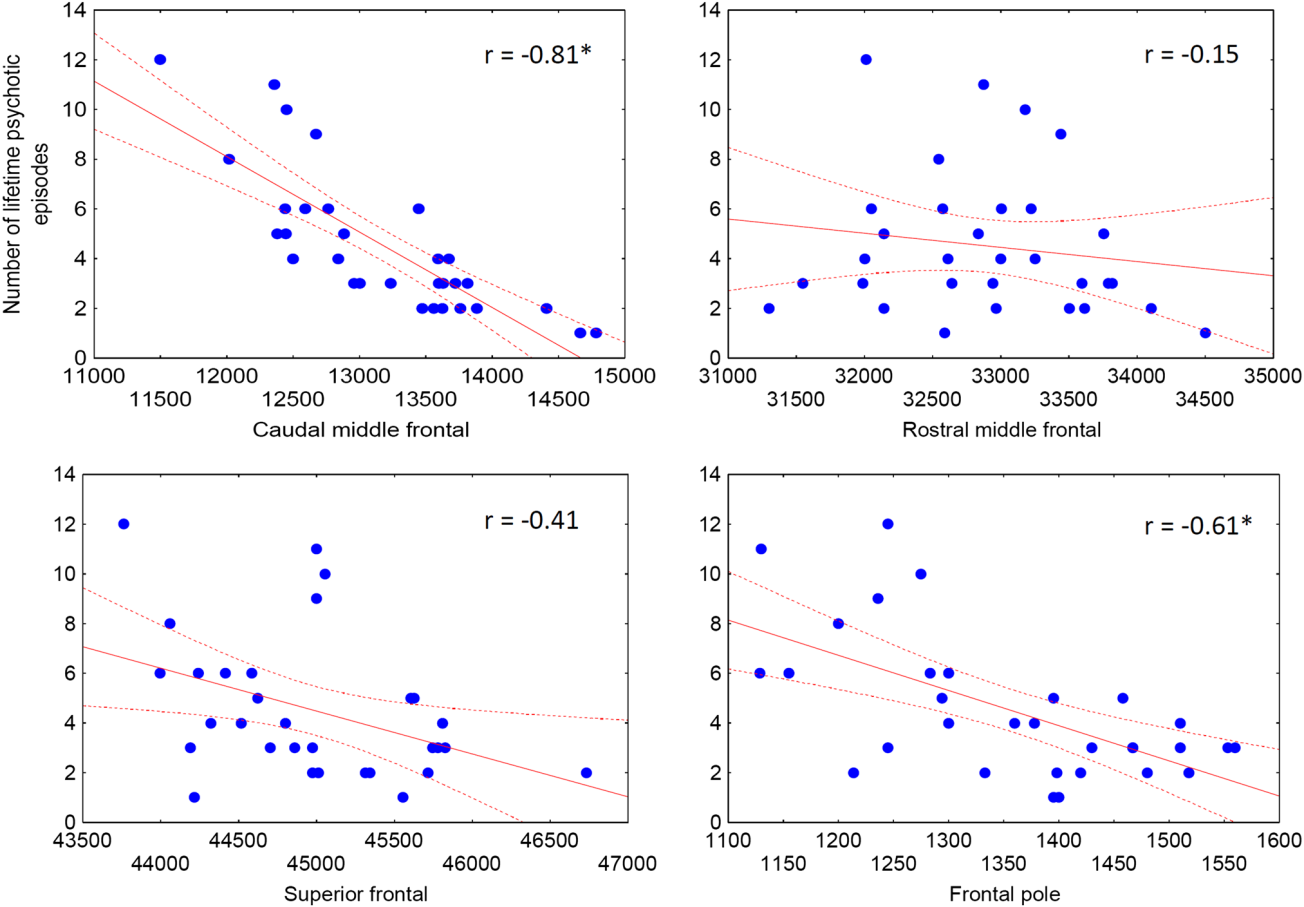
Correlations between frontal volumes (mm^3^) and the number of lifetime psychotic episodes in patients with brief psychotic disorder (BPD)

There were no significant correlations between brain volumes, PANSS, lifetime antipsychotic dose-years, duration of illness in years, and age (− 0.2 < *r*s < 0.2, *p*s > 0.2). We found no significant differences between medicated (*n* = 8) and unmedicated (*n* = 22) BSD patients (*p* > 0.5). The number of lifetime psychotic episodes negatively correlated with caudal middle frontal volumes (*r* = − 0.81, *p* < 0.001) and frontal pole volumes (*r* = − 0.61, *p* < 0.001) (Fig. 1.).

## Discussion

We observed a selective volume reduction in the frontal cortex in BPD, including the caudal/ rostral middle frontal, superior frontal, and frontal polar regions. Smaller frontal volumes were associated with one of the most important clinical features of BPD, that is, the number of recurrent psychotic episodes without conversion to schizophrenia or other long-lasting psychotic disorders. Certainly, correlation analyses do not provide information on the direction of causality. It is not clear whether a pre-existing decrease in frontal volumes predisposes to recurrent psychotic episodes, or frequent psychotic breakdowns result in cortical volume loss.

The results of the present study are different from that documented in schizophrenia (Fornito et al. 2009; Haijma et al. 2013; Honea et al. 2005; Shepherd et al. 2012; Wright et al. 2000): we did not find smaller volumes in temporal, parietal, subcortical, medio-temporal, and other limbic regions in BPD. Given the paucity of neuroimaging studies in BPD, comparisons with previous results are difficult. Early computed tomography (CT) studies from cycloid psychosis, which is characterized by short psychotic episodes that does not necessarily fit into any DSM-5 category, provided inconsistent results regarding ventricular abnormalities (Hoffler et al. 1997; Franzek et al. 1996). It is possible that circumscribed frontal alterations in BPD are a "tip of the iceberg” phenomenon, and a more extensive volume reduction could be detected in a larger sample of BPD patients. Methodological differences (e.g., FreeSurfer regional volumetry vs. voxel-based morphometry) are also critical, which may lead to the heterogeneity of results (Honea et al. 2005; van Erp et al. 2018). In an extensive meta-analysis with standardized FreeSurfer methodology, including thousands of patients with schizophrenia and controls from 39 centers, van Erp et al. (2018) demonstrated a widespread cortical thinning in patients, with the largest negative effect sizes in bilateral fusiform, temporal (inferior, middle, and superior), and left superior frontal gyri, right pars opercularis, and bilateral insula. Moreover, schizophrenia is associated with a progressive loss of cortical gray matter, and this reduction may be due to antipsychotic treatment, especially in the case of first-generation drugs (Haijma et al. 2013; Fusar-Poli et al. 2013; Vita et al. 2015; Olabi et al. 2011). Honea et al. (2005) concluded that brain loss in schizophrenia may be explained by a combination of early neurodevelopmental processes and illness progression. The pathophysiology of progressive changes in brain structure is unknown. In addition to the negative effect of high-dose first-generation antipsychotics, intrinsic mechanisms of illness progression (e.g., glutamatergic dysfunction, decreased neurotrophic factors, and neuroinflammation) may also play a role (Bauer and Teixeira 2019). It is unclear whether these schizophrenia-related mechanisms are also implicated in BPD. By reviewing data from electrophysiology and brain metabolism, Malhotra et al. (2019) concluded that acute and transient psychoses are characterized by hyperarousal and hypermetabolism, whereas schizophrenia is associated with hypoarousal and hypometabolism. Increased peripheral inflammatory markers have also been detected in transient psychosis (Malhotra et al. 2019). In addition, we found evidence for a circumscribed volume loss in the frontal cortex, which did not correlate with illness duration or cumulative lifetime antipsychotic use in BPD. It is important to bear in mind that the present study was cross-sectional, and, therefore, our data did not provide direct evidence of progressive brain changes in BPD. In addition, separate groups of patients with recent-onset BPD and schizophrenia were not included in this study, and the direct comparison of these disorders is not possible.

The most important limitation of the present study is the small sample size. Therefore, the results must be replicated in a large sample from multiple clinical centers. Due to the small sample size and the issue of multiple comparisons (type I errors), we did not conduct separate analyses for the left and right hemispheres. Another limitation is that we did not apply structured clinical interviews for personality disorders. It is a relevant and important issue because the prevalence of personality disorders is high in acute and transient psychoses (Jorgensen et al. 1996), and it may affect brain structure, especially in the case of schizotypal and borderline personality disorder. However, structural abnormalities in the frontal cortex are not characteristic for schizotypal (Fervaha and Remington 2013) and borderline personality disorder (Kimmel et al. 2016).

Prefrontal dysfunctions are probably the most widely researched area in psychotic disorders, which may be implicated in the developmental and neurocognitive mechanism of psychosis (e.g., executive dysfunctions, abnormal self-monitoring, and social-cognitive impairments) (Cannon 2015; Tan et al. 2009; Sakurai and Gamo 2019). The main conclusion of the present study is that prefrontal dysfunctions are also important in BPD. Future studies should explore the functional, neuropsychological, and neurochemical correlates of these deficits. These multidisciplinary investigations are warranted because of the severe unmet needs of patients with short-lived psychotic episodes. For example, in a large sample of patients with a first index diagnosis of acute and transient psychosis, a 8-year follow-up study indicated that almost two-third of the patients (61.3%) retained the index diagnosis, whereas the remaining individuals developed persistent psychotic disorders (Rutigliano et al. 2018). The majority of individuals suffering from transient psychosis are invisible for high-risk and early intervention services, despite the fact that the clinical and psychosocial outcomes are severe, and clinical treatment approaches are heterogeneous and not satisfactory (Minichino et al. 2019). Thus, a more detailed investigation and characterization of the neurological phenotype and mechanisms are necessary to refine the diagnosis and treatment strategy in patients with BPD.
